# SLAMF1 contributes to cell survival through the AKT signaling pathway in Farage cells

**DOI:** 10.1371/journal.pone.0238791

**Published:** 2020-09-04

**Authors:** Heejei Yoon, Eung Kweon Kim, Young Hyeh Ko

**Affiliations:** 1 Clinical Medicine Research Center, School of Medicine, Sungkyunkwan University, Seoul, South Korea; 2 Department of Ophthalmology, Corneal Dystrophy Research Institute, College of Medicine, Yonsei University, Seoul, South Korea; 3 Department of Pathology, Samsung Medical Center, School of Medicine, Sungkyunkwan University, Seoul, South Korea; Marshall University, UNITED STATES

## Abstract

SLAMF1 is often overexpressed in Epstein Barr virus (EBV)-infected B cell tumors. However, its role in the pathogenesis of EBV-infected B cell tumors remains largely unknown. Here, we generated SLAMF1-deficient EBV+ tumor cells and examined the effect of its deficiency on cell proliferation and cell survival. There were no significant differences in cell proliferation and cell cycle distribution for short periods between the SLAMF1-deficient and wild-type cells. However, the deficient cells were more resistant to an AKT inhibitor (MK-2206). When the both cells were co-cultured and repeatedly exposed to the limitations in nutrition and growth factors, the SLAMF1-deficient cells were gradually decreased. We observed that levels of phospho-AKT were differentially regulated according to the nutritional status between the SLAMF1-deficient and wild-type cells. A decrease in phospho-AKT was observed in SLAMF1-deficient cells as well as an increase in pro-apoptotic Bim just before cell passage, which may have been due to the loss of SLAMF1 under poor growth condition. Overall, SLAMF1 is not a strong survival factor, but it seems to be necessary for cell survival in unfavorable growth condition.

## Introduction

Signaling lymphocyte activation molecule family member 1 (SLAMF1/CD150) is a self-ligand receptor that is expressed on activated T- and B-lymphocytes, dendritic cells and macrophages [1, 2]. Ligation of SLAMF1 on B cells enhances cell proliferation and apoptosis triggered by the activation of CD40 and Fas/CD95, respectively [3, 4]. SLAMF1 also serves as a receptor for measles virus and a phagocytic element for bacterial killing [1, 5–7]. SLAMF1 was differentially expressed according to B cell developmental stages [8], NF-κB activation [9], or the cell of origin [10]. For instance, it was positive in activated B-cell-like (ABC) DLBCL, but not in germinal center B-cell-like (GCB) DLBCLs [10]. SLAMF1 is highly induced in the Epstein Barr virus (EBV)-infected lymphoblastoid cell lines (LCL) and the latency type III EBV+ lymphoma [9, 11]. We reported the overexpression of SLAMF1 and recurrent copy number gain at 1q23.3 harboring SLAMF1 in EBV+ DLBCL [12]. This suggests that SLAMF1 might play a role in the pathogenesis of EBV+ DLBCL.

SLAMF1 is a type I glycoprotein that consists of two Ig-like extracellular domains and a cytoplasmic tail. Its cytoplasmic domain contains two immunoreceptor tyrosine-based switch motifs (ITSMs) (TxYxxI/V) [1]. These switch motifs interact with several molecules that initiate cell signaling. They include adaptor molecules SH2D1A (SAP) and SH2D1B (EAT-2), the phosphatases SHP-2 and SHIP and the Src-family kinases Fyn, Lyn, and Fgr [1]. Positive or negative signaling through SLAMF1 depends on type of molecules bind to it. Ligation of SLAMF1 could activate the ERK and AKT signaling pathways [10, 13]. In tumor cells, ligation of SLAMF1 resulted in transient phosphorylation of Akt or dephosphorylation depending on cellular context [10]. However, the significance of the activation of these signaling pathways remains to be explored.

To understand the possible role of SLAMF1 in EBV+ DLBCL, we depleted SLAMF1 in EBV+ Farage cells and tested its effect on cell proliferation and survival. SLAMF1 was not necessary for cell survival when cultured in optimal growth conditions, but it might be necessary under unfavorable growth condition.

## Materials and methods

### Cell culture and reagents

Farage cells are an EBV+ B cell lymphoma cell line, which were purchased from ATCC (American Type Culture Collection, Manassas, VA, USA) and cultured in RPMI 1640 media containing 10% fetal bovine serum (FBS) and antibiotics (100 μg/mL penicillin and streptomycin) at 37°C under a 5% CO_2_ humidified atmosphere. Cell line authentication and mycoplasma testing were performed periodically in our institute.

### Generation of SLAMF1-deficient Farage cells

SLAMF1-deficient Farage cells were generated by using the Guided it-CRISPR/Cas9 System (Clontech Laboratories, Inc., Mountain View, USA). Oligomers for custom sgRNAs that target exon3 of SLAMF1 were designed using on-line tools (http://crispr.mit.edu/) and cloned into the pGuide-it vector according to company’s manual.

The sequences of sgRNA were as follows: 5'- CG GGA CCT GCA CCT TGA TAC-3' and 5'- AT TCC CAG ACC TTC AGC CCG-3'. The cloned vectors were mixed with T buffer (Lonza, Allendale, NJ, USA) and transfected into Farage cells by electroporation with Nucleofector (Lonza, program S-018). Farage cells expressing Cas9 were selected using FACS Aria III (BD Life Sciences, San Jose, CA, USA) 2 days after electroporation and were cloned by serial dilution and FACS-mediated selection. Insertion or deletion mutations were screened with GeneArt Genomic Cleavage Detection kit (Thermo Fisher Scientific, Waltham, MA, USA) after PCR amplification of exon 3. The primer sequences for PCR were as follows: EX3-fw, 5'-CCA TGT GAA GAC TGA GCC CAT G-3'; EX3-rev, 5'-CCA GGG GTT CAC TTC AGT GAT G-3'. To identify nonsense or missense mutations, the PCR products were directly sequenced or cloned using TOPO TA cloning kit and sequenced.

### Fluorescence-activated cell sorting (FACS) analysis

SLAMF1-deficient and wild-type Farage cells were seeded at a density of 2 × 10^5^/mL cells in T25 flasks and cultured for 2 days. Then, 1 × 10^6^ cells were then washed, and their Fc receptors were blocked with TruStain (Biolegend, San Diego, CA, USA). Cells were labeled with FITC-conjugated IgG or anti-human SLAMF1/CD150 antibody (Cat# 306306, Biolegend, San Diego, CA, USA) and were subject to fluorescence-activated cell sorting (FACS) analysis by a Becton-Dickinson FACS Calibur (Franklin Lakes, NJ, USA).

### Drug inhibition experiments

SLAMF1-deficient and wild-type Farage cells were seeded at a density of 2 × 10^4^ cells in 96-well plates. Cells were treated with various concentrations of MK-2206 (Selleckchem, Houston, TX, USA) for 3 days. Cell proliferation and viability were measured using the WST-1 reagent according to the manufacturer's protocols (Roche, Indianapolis, IN). Optical density was measured at 450 and 600 nm 4 h after the addition of the WST-1 reagent. The concentration to reduce cell viability by 50% (IC_50_) was calculated using package drc (http://cran.r-project.org/web/packages/drc/). We used the 3-parameter logistic function standard curve analysis for dose response.

### Cell proliferation and cell cycle analysis

Cell proliferation was accessed by counting viable cells at 1, 3, and 5 days after seeding at 1 × 10^5^/mL cells. For the cell cycle analysis, Farage cells were washed with cold 1 × PBS and then fixed with 70% cold ethanol for more than 30 min at -20°C. Cells were washed with 1 × PBS and incubated with a staining solution including 50 μg/mL propidium iodide (PI) and 200 μg/mL RNase A for 15 min at 37°C. DNA content was analyzed using a Becton-Dickinson FACS Calibur flow cytometer. Data were analyzed with CellQuest software (Becton Dickinson, Heidelberg, Germany).

### Immunoblot analysis

The SLAMF1 wild-type and mutant Farage cells were lysed with M-PER buffer (Pierce Biotechnology, Rockford, IL) containing a 1× protease and phosphatase inhibitor cocktail (Roche). Then, 20–40 μg of lysate was separated on 4–15% or 12% precast sodium dodecyl sulfate-polyacrylamide gels and transferred onto polyvinylidene fluoride membranes (Bio-Rad Laboratories, Hercules, CA). The membranes were blocked with 5% non-fat dry milk and incubated with appropriate primary and secondary antibodies. Signals were detected using the SuperSignal West Pico Chemiluminescent Substrate (Pierce Biotechnology). The following antibodies were used in this study: phospho-NF-kB p65 (3033), BCL-XL (2764), MCL1 (5453), Bcl-2 (4223), Bim (2933), HSP70 (4782), phospho-AKT (4060) and panAKT (4691) were purchased from Cell Signaling Technologies (Beverly, MA); LMP1 (CS1-4, M0897) from Dako (Glostrup, Denmark); GAPDH (sc-25778), and goat anti-rabbit IgG (sc-3837) from Santa Cruz Biotechnology (Santa Cruz, CA, USA). We developed an anti-SLAMF1 rabbit polyclonal antibody for western blotting.

### Analysis of apoptosis

SLAMF1 mutant and wild-type Farage cells were seeded at 2×10^5^ cells/mL and cultured for 5 days. Cells were washed with 1× PBS and stained with fluorescein isothiocyanate (FITC)-conjugated anti- Annexin V antibody (BD Biosciences, Heidelberg, Germany) and propidium iodide (PI) (Sigma Aldrich, St. Louis, MO, USA). Detection of early apoptotic (Annexin V-FITC+ and PI-) and late apoptotic cells (Annexin V-FITC+ and PI+) was performed using a FACS Calibur (Becton and Dickinson). Data were analyzed with CellQuest software (Becton Dickinson).

### Statistical analysis

All data were analyzed with the statistical program GraphPad Prism 6.0 (San Diego, CA). Student’s t-test was used to determine the statistical significance of differences between groups.

## Results

### Generation of SLAMF1-deficient Farage cells

**Fig 1:**
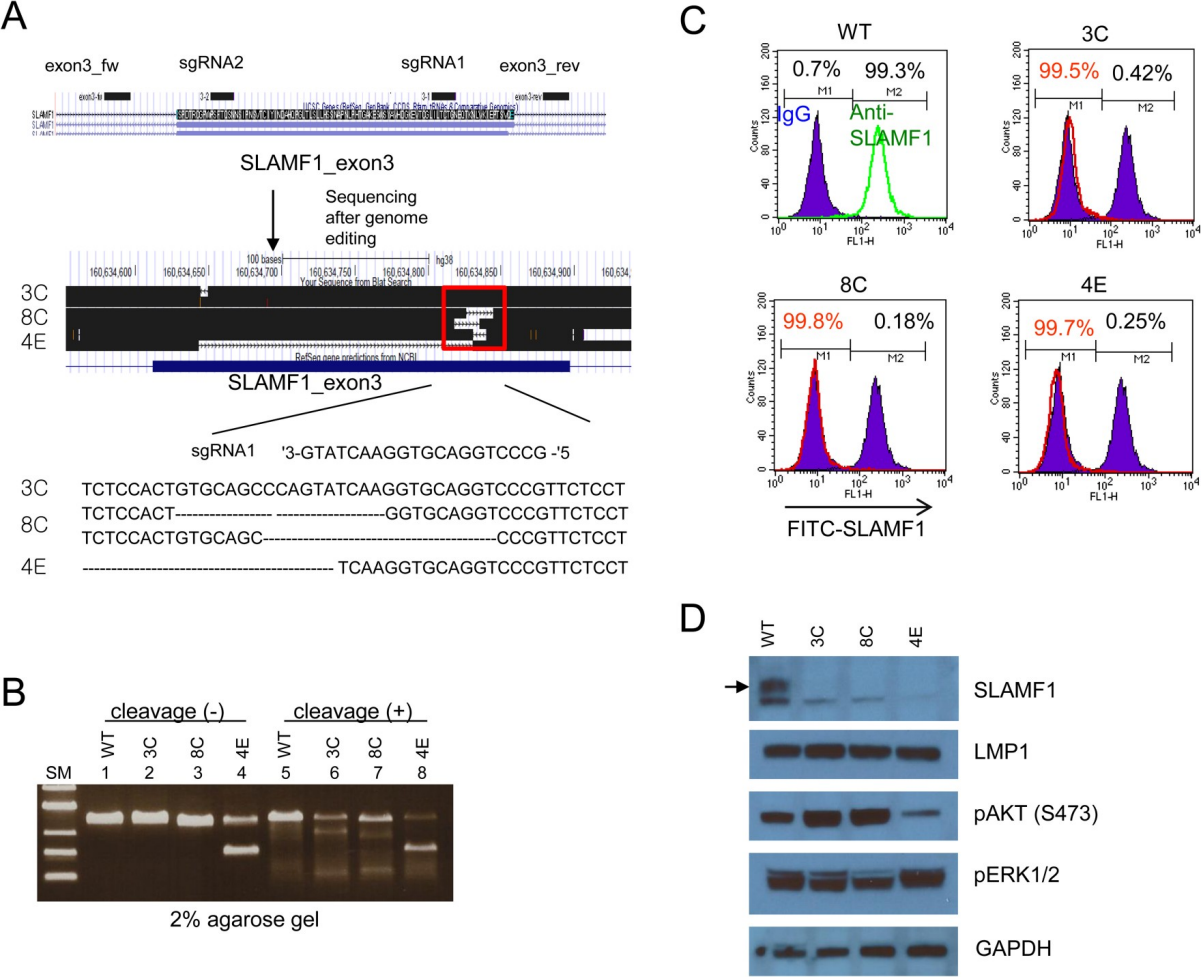
SLAMF1-deficient Farage cells were cloned by using CRISPR/Cas9. (A) The two regions on exon 3 indicate two sgRNA sites of SLAMF1. The two regions outside of exon 3 indicate two primer sites for PCR amplification (upper panel). Indel mutations were confirmed with sequencing and are depicted in the lower panel. (B) Genomic DNA isolated from the selected clones was PCR-amplified. The PCR products were denatured and digested after allowing for the formation of duplexes. Cleaved DNAs were analyzed on 2% agarose gel. (C) SLAMF1 on the cell surface of wild-type and mutant Farage cells were analyzed by FACS. The left peak indicates Farage cells labeled with FITC-anti-IgG antibody, whereas a right peak indicates Farage cells labeled with a FITC-anti-SLAMF1 antibody. The SLAMF1 mutant Farage cells overlap with the left peak (depicted in red). (D) A depletion of SLAMF1 was confirmed by western blot for SLAMF1, LMP1, phosphor-AKT (S473) and GAPDH.

SLAMF1 was highly expressed in Farage cells, which is an EBV positive B cell lymphoma cell line. To generate SLAMF1-deficient Farage cells, we designed two sgRNAs targeting exon 3 of SLAMF1 by using CRISPR/Cas9 technology (Fig 1A). The two sgRNAs coupled with Cas9 expression vectors were transfected, and three cell lines named 3C, 8C and 4E were obtained by serial dilution and mutational analysis (Fig 1B). As shown in Fig 1B, two bands were observed in 4E after PCR amplification, indicating a deletion of exon 3 in one allele (Fig 1B). Sub-bands appeared after cleavage of heteroduplex in 3C, 8C and 4E, indicating the presence of at least one indel mutation (Fig 1B). We next tested whether expression level of SLAMF1 was decreased at the cell surface by using FACS analysis. SLAMF1 was almost negative in the three cell lines compared with SLAMF1 wild-type Farage cells (Fig 1C). This result was further confirmed by western blot analysis with the anti-human SLAMF1 antibody, which was a different clone than that used for FACS analysis. SLAMF1 was not detected or remarkably decreased in mutant cell lines, whereas LMP1 had no discernable change between SLAMF1 wild-type and mutant cells (Fig 1D). Of note, phospho-ATP was variable between them, suggesting that the deficiency of SLAMF1 disturbs the AKT signaling pathway.

### Differential response to an AKT inhibitor

**Fig 2:**
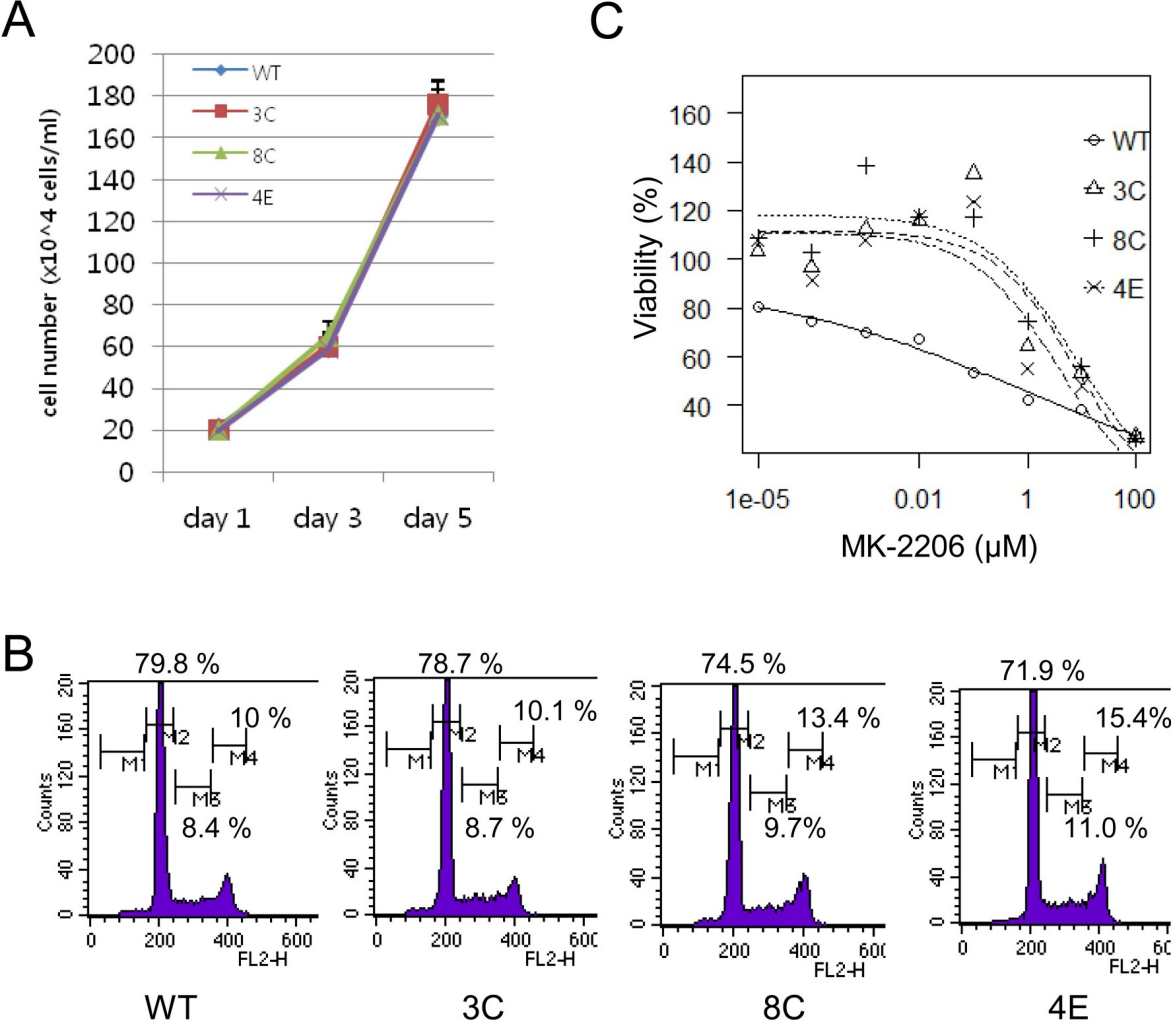
Comparison of SLAMF1 wild-type and SLAMF1-deficient cell proliferation. (A) The effect of SLAMF1 on cell proliferation was assessed by counting viable cells for 5 days. (B) The cell cycle distribution was analyzed by using FACS. Cells fixed with 70% ethanol were stained with propidium iodide (PI) and subject to FACS analysis based on the DNA content. (C) Cells were incubated with various concentrations of AKT inhibitor MK-2206 for 3 days. Cell proliferation was assessed with WST-1 reagent. The Y-axis indicates viability (%). IC_50_ of the 3C, 8C, 4E and WT cells were 7.6, 7.9, 3.7 and 1.05 μM.

SLAMF1 was implicated as a co-stimulatory molecule for cell proliferation and cell death. Therefore, we performed cell proliferation assay by counting cells for 5 days (Fig 2A). There was no significant difference in cell proliferation among the four cell lines. Next, cell cycle analysis was performed by using FACS. Similarly, there was no significant difference in cell cycle distribution among the cell lines (Fig 2B). We observed overexpression of SLAMF1 at the cell surface of drug resistant Farage cells [14]. We therefore tested whether SLAMF1-deficient Farage cells responded differentially to drugs targeting several pathways. A differential response was observed only in cells treated with an AKT inhibitor (MK-2206) among the drugs tested. For MK-2206, IC_50_ of the mutant cells 3C, 8C and 4E were 7.6, 7.9 and 3.7 μM, respectively, whereas IC_50_ of WT cells were 1.05 μM (Fig 2C). The mutant cells exhibited more resistance to MK-2206 compared with WT cells. In addition to variable phospho-AKT levels (Fig 1D), this result suggested that loss of SLAMF1 might affect the AKT signaling pathway.

### Loss of SLAMF1 affects AKT and Bim

**Fig 3:**
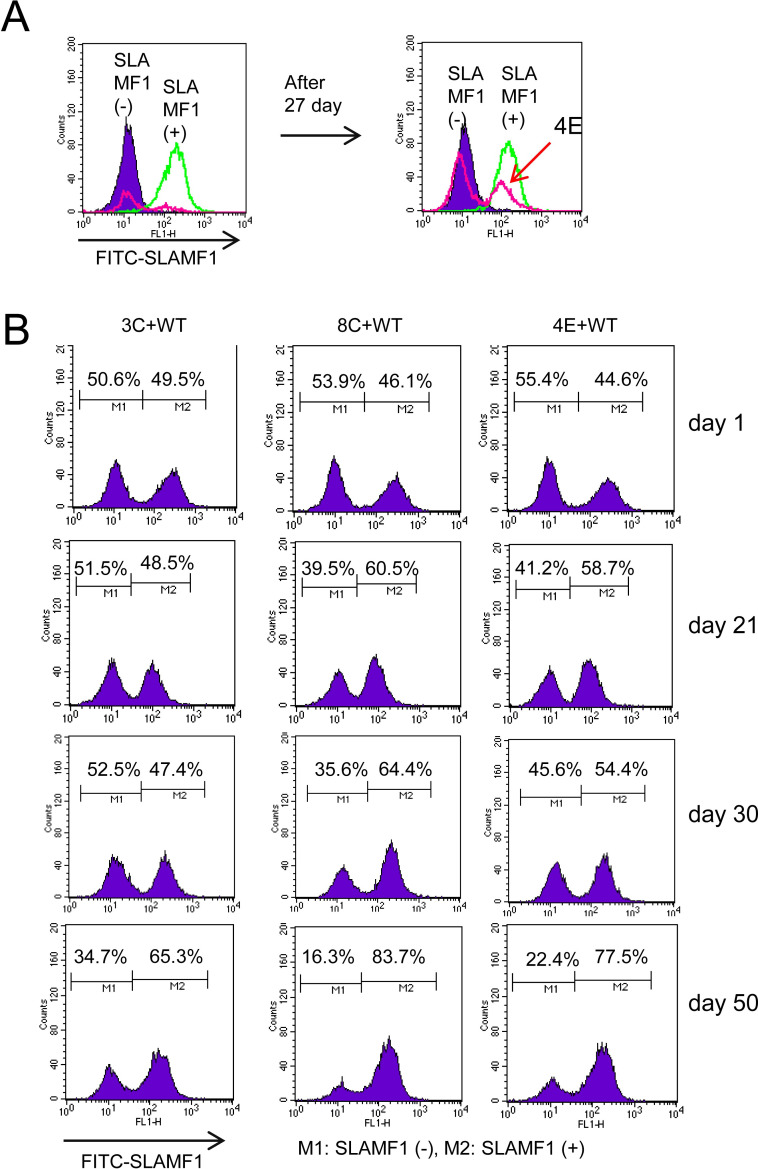
Outgrowth of SLAMF1 wild-type cells over mutant cells during long-term co-culture. (A) Proportion of wild-type cells that were gradually increased when 4E cells had been contaminated with wild-type cells at the initial cloning stage. (B) SLAMF1 wild-type cells and mutant cells were mixed at a 1:1 ratio at day 0. During the first one month, the cells were subcultured every 5 days, and then the next month, the cells were subcultured every 7 days. Wild-type cells became dominant in all three mutant cell lines as the interval between cell passages increased.

**Fig 4:**
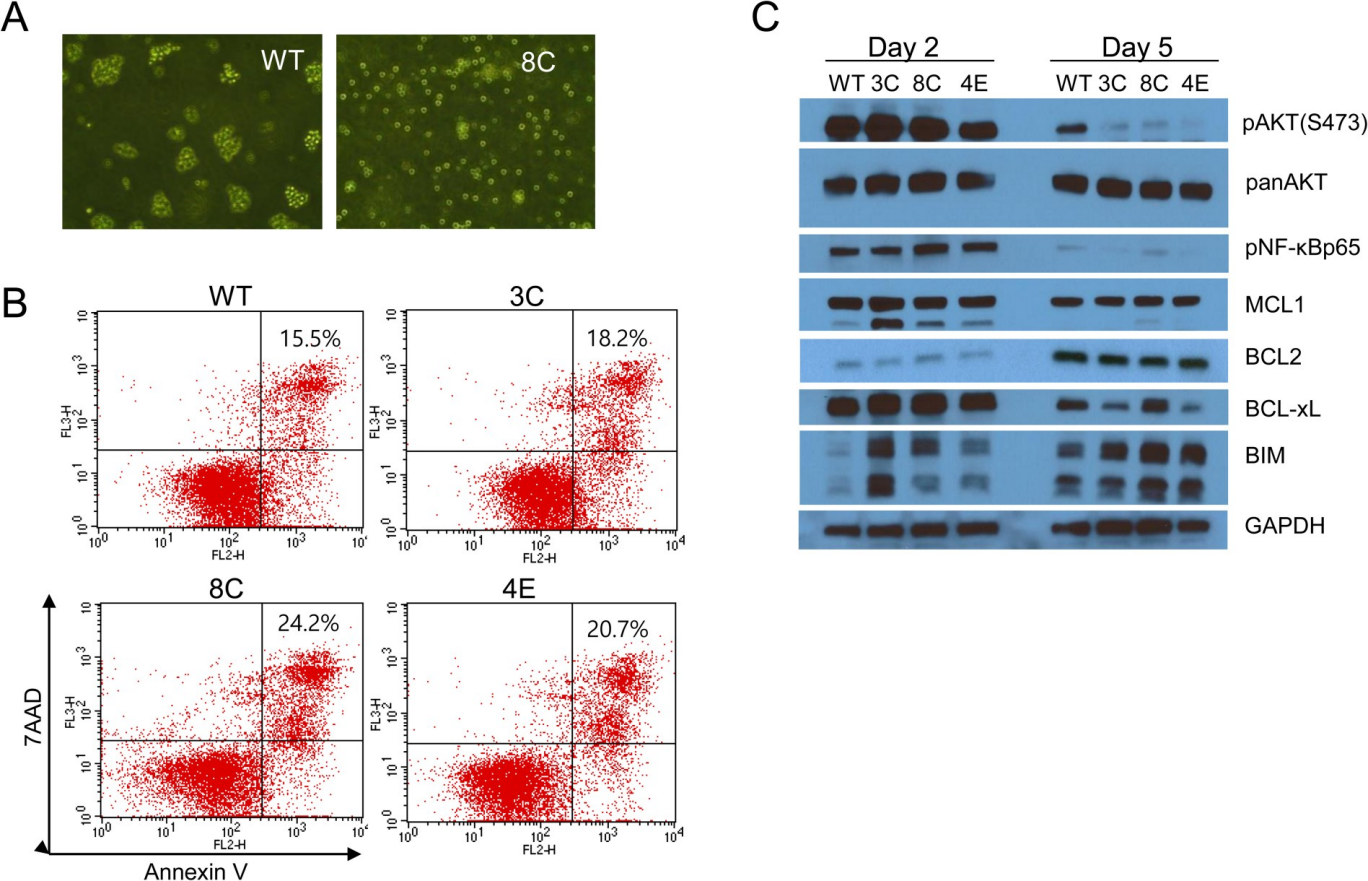
Increase of Bim might underlie slightly increased apoptosis in SLAMF1 mutant cells at day 5. (A) Cell images of SLAMF1 wild-type and mutant (8C) cells at day 5. Wild-type cells were kept aggregated together, whereas mutant cells became separated. (B) Analysis of apoptotic cells using FACS after staining cells with anti-Annexin V and propidium iodide (PI). Double positive region (upper right) indicates late apoptotic cells (Annexin V-FITC+ and PI+). (C) SLAMF1 mutant and wild-type Farage cells were seeded at 2×10^5^ cells/mL and lysed 2 and 5 days after culture. Western blotting for phospho-AKT (S473), phospho-NF-kB p65, BCL2, MCL1, BCL-xL, and Bim at days 2 and 5.

When we were trying to clone the SLAMF1-deficient Farage cells, we observed that SLAMF1 wild-type Farage cells often out-competed the SLAMF1-deficient cells (Fig 3A). Therefore, we mixed both wild-type and mutant SLAMF1 Farage cells and cultured them together for more than one month with repeated passages every 5 and 7 days. One mutant cell (8C) was gradually out-competed after the 5-day cell passages for one month, whereas two other mutant cells (3C and 4E) were more clearly out-competed by the 7-day cell passages by wild-type cells, which then dominated the mixed cell population (Fig 3B). We observed that before the cell passages wild-type cells were kept aggregated together, whereas a part of the SLAMF1-deficient cells became separated (Fig 4A). We hypothesized that some SLAMF1-deficient cells might be less tolerant to nutritional deficiency or acidic microenvironment and undergo programmed cell death. We analyzed apoptotic cells with FACS after labeling cells with Annexin V plus 7-AAD. We found that late apoptotic cells were increased in all SLAMF1-deficient cells (Fig 4B). We then performed a western blot analysis for phospho-AKT and anti- or pro-apoptotic genes at 2 and 5 days after passaging. Interestingly, phospho-AKT was higher in SLAMF1-deficient cells than in wild-type cells at day 2 (3C and 8C) (Fig 4C), whereas it was much lowered at day 5 (Fig 4C). The level of phospho-NF-kB p65 was greatly decreased at day 5 when compared to day 2, but no apparent differences were seen between wild-type and mutant cells. The pro-apoptotic gene Bim seemed to be counterbalanced by MCL1 and Bcl-XL as shown in the 3C line at day 2. Bim was increased in SLAMF1-deficient cells without an increase in MCL1 at day 5. The balance between anti- or pro-apoptotic genes seemed to shift towards pro-apoptosis due to the increased Bim at day 5, which might be responsible for the increased apoptosis in the mutant cells.

## Discussion

In this study, we observed that under optimal growth conditions, a deficiency of SLAMF1 made no discernible differences in cell proliferation and cell cycle distribution in EBV+ Farage cells. However, when the Farage cells were exposed to limited nutrition and growth factors, SLAMF1 was required to evade apoptosis through the activation of AKT.

Evading apoptosis is one of the characteristics acquired by cancer cells [15]. EBV suppresses apoptosis of EBV-infected B cells by several mechanisms. First, the EBV latent membrane protein LMP1 activates classical or non-classical NF-κB pathway, which results in an increase of the anti-apoptotic gene Bcl-xL and survivin [16]. Secondly, EBV down-regulates the pro-apoptotic gene Bim. EBV-infected B cells down-regulate Bim [17]. Especially, EBV nuclear proteins EBNA3A and 3C repress the transcription of Bim by recruiting polycomb repressive complex 2 (PRC2) [18] and chromatin remodeling. EBV’s clustered multiple miRNAs attenuate Bim [19]. Bim was significantly downregulated in EBV-positive compared to EBV-negative posttransplant lymphoproliferative disorders (PTLD) [20]. The reason why Bim is a target of EBV is that Bim is responsible for cell death by depriving survival factors as demonstrated in many cells originating from the hematopoietic lineage [21–26]. Therefore, loss of Bim renders cells resistant to apoptosis induced *in vitro* by cytokine or growth factor deprivation even in a B cell lymphoma model [27]. In Fig 4, we observed increased Bim levels in SLAMF1-deficient cells at day 5 when cytokines or growth factors were not be sufficient for survival, but not in SLAMF1 wild-type Farage cells. We also observed a reverse relationship between phospho-AKT and Bim, which provided clues for how Bim might be regulated in Farage cells [22, 28]. The activated AKT phosphorylates FOXO3, which renders FOXO3 sequestered in cytosol, thereby inhibiting transcription of a pro-apoptotic gene Bim by FOXO3. Taken together, we speculate that SLAMF1 regulates the AKT pathway, which leads to suppression of Bim to evade apoptosis under unfavorable growth conditions.

SLAMF1 was known to costimulate proliferation of activated B cells [3]. In a previous study [14], we used SLAMF1 as a surrogate marker for LMP1 and observed that the SLAMF1^high^ cells grew faster than the SLAMF1^low^ cells. However, loss of SLAMF1 itself did not obviously alter cell proliferation and cell cycle distribution at the exponential growth phase. This indicated that other factors such as LMP1, which is epistatic to SLAMF1, play a major role in the proliferation of Farage cells. Unexpectedly, we repeatedly observed that the SLAMF1 mutant cells (3C and 8C) proliferated faster in the presence of an allosteric AKT inhibitor (MK-2206) at a low dose compared to SLAMF1 wild-type cells (Fig 2C). We cannot fully explain this result; however, different levels of activated AKT might explain the underlying mechanism of this result. The greater amount of activated AKT was seen in 3C and 8C mutant cells compared to wild-type cells at day 2 (Figs 1 and 4). Therefore, MK-2206 might not fully inhibit AKT phosphorylation at the same amount that can inhibit AKT in wild-type cells. This result implies that SLAMF1 might down-regulate the AKT pathway under optimal growth conditions.

The relative amount of phospho-AKT in SLAMF1 mutant vs. wild type cells was reversed between days 2 and 5 in our experiment (Fig 4). It was questioned how SLAMF1 functions as a costimulatory molecule in opposite cellular events of cell proliferation and apoptosis [11]. This contradictory function is thought to be attributable to its cytoplasmic domain with two ITSM that interact with several molecules such as the adaptors SAP and EAT-2, SHIP, SHP-1, Lyn, and PI3K [1, 10]. Although the molecules that are recruited to these motifs at days 2 and 5 have not yet been identified, SLAMF1 might recruit phosphatases such as SHP-2 and SHIP or kinases to its cytoplasmic tail to maintain optimal strength of the AKT signaling pathway required for proliferation and survival in Farage cells. In conclusion, SLAMF1 is not a strong survival factor, but it might be necessary for cell survival under unfavorable growth condition.

## Supporting information

S1 Raw images (PDF)
