# Supplementary material for: SLAMF1 contributes to cell survival through the AKT signaling pathway in Farage cells
Source: PLoS One. 2020 Sep 4;15(9):e0238791. doi: 10.1371/journal.pone.0238791 (PMC7473542; doi:10.1371/journal.pone.0238791)
Supplement: S1 Raw images — (PDF) [file pone.0238791.s001.pdf]

Figure 1D in manuscript

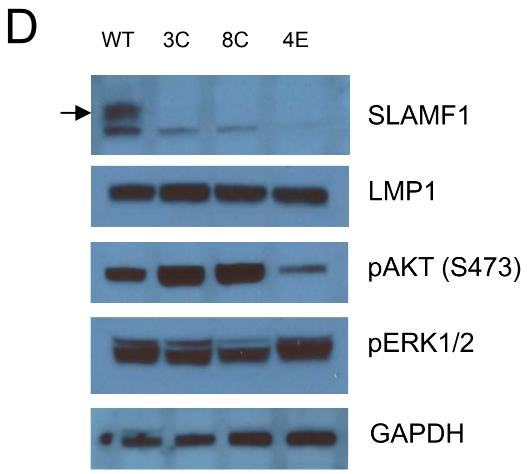

Raw data

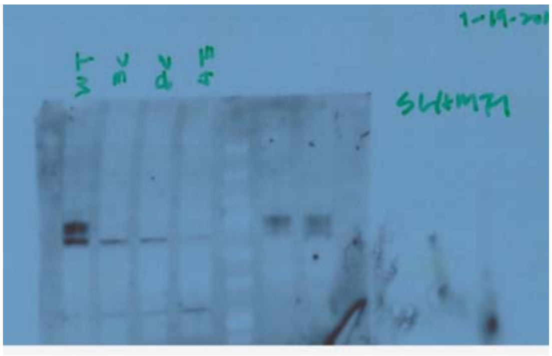

western blot: SLAMF1

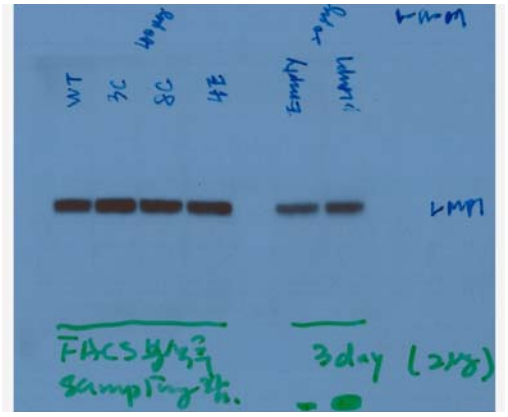

western blot: LMP1

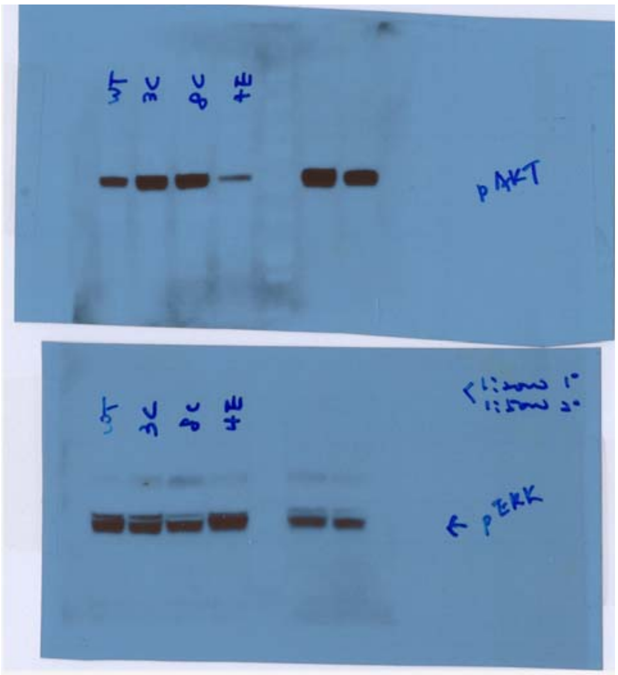

western blot: p-AKT and p-ERK

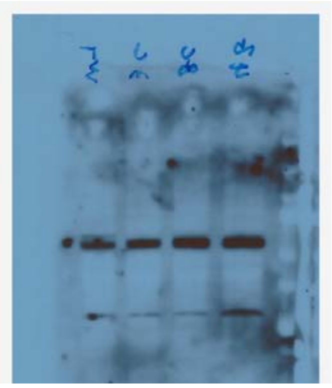

western blot: GAPDH

Figure 4C in manuscirpt

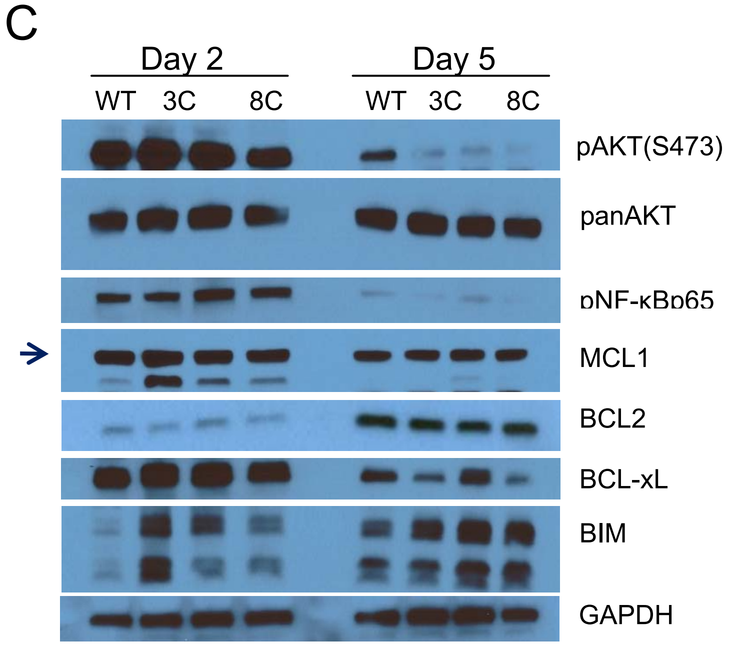

Raw data

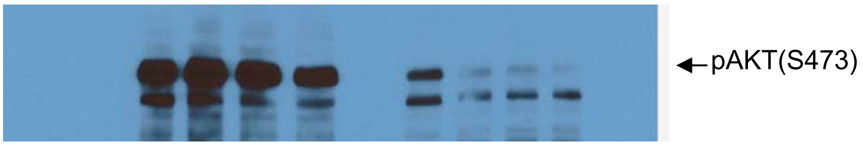

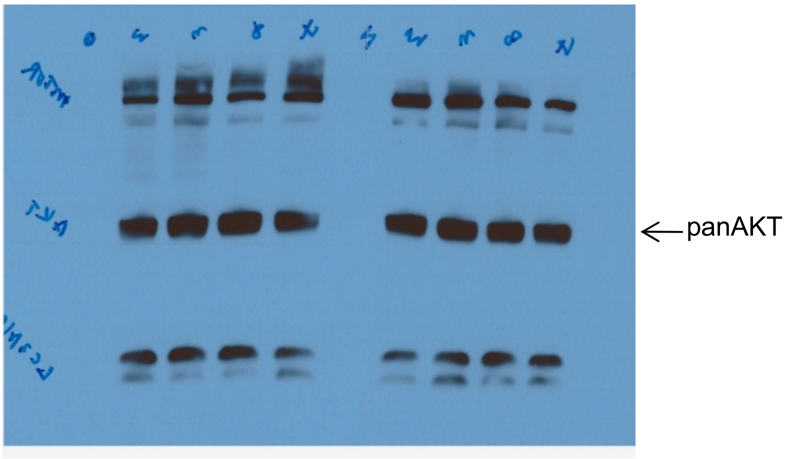

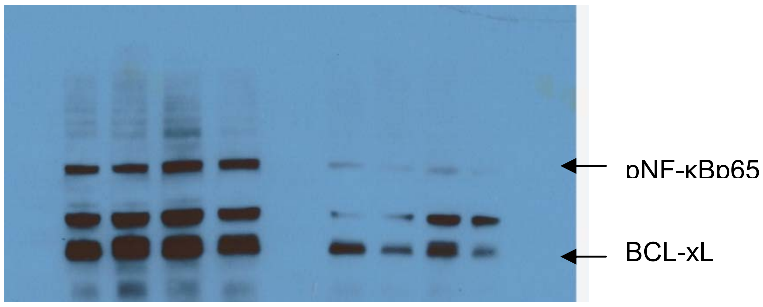

pNF-κBp65

BCL-xL

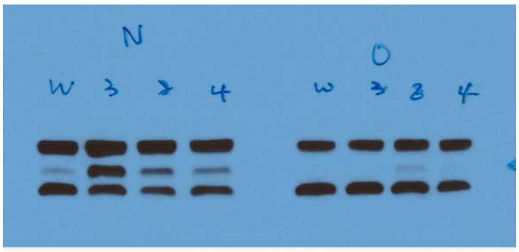

MCL1

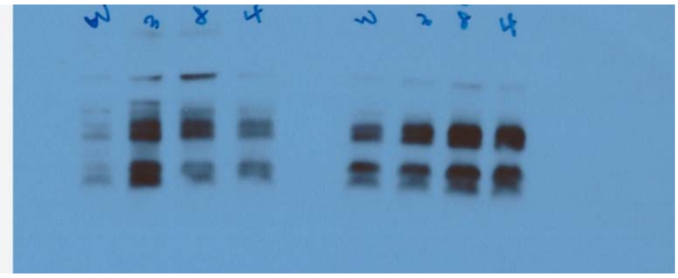

BIM

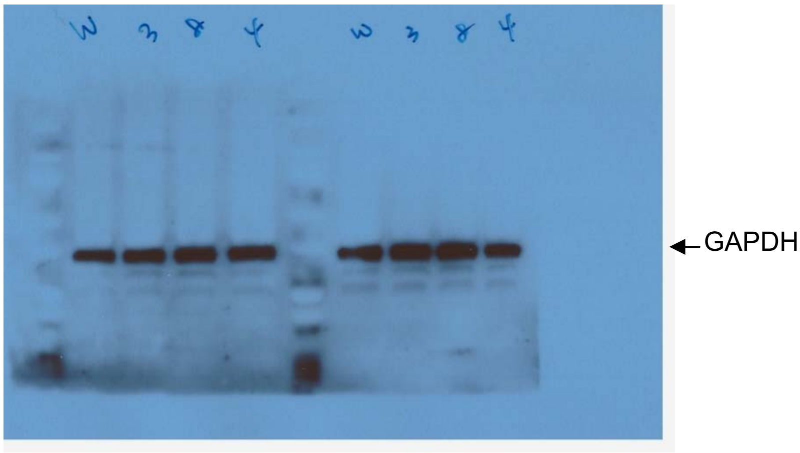

GAPDH

Repeat western blot: MCL1, pAKT(S473), BCL2, BIM

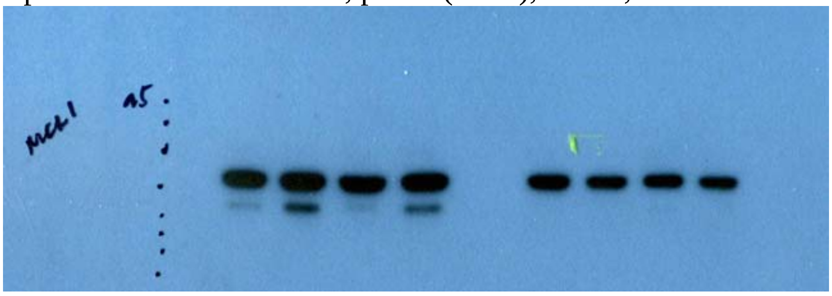

MCL1

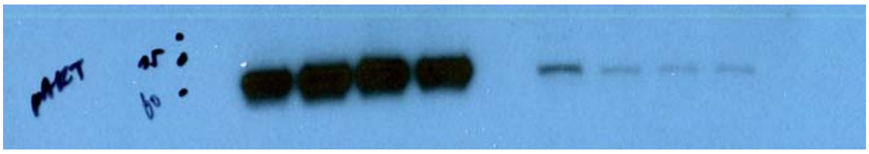

pAKT(S473)

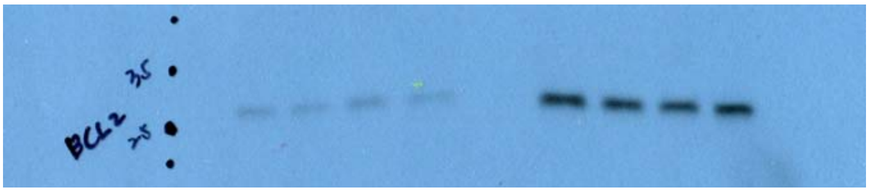

BCL2

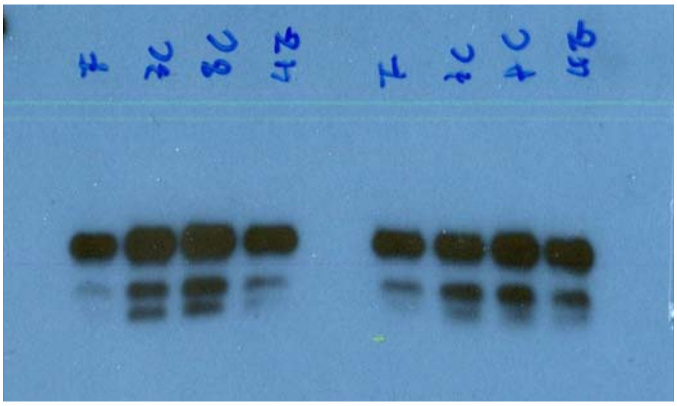

BIM
